# Soy Isoflavones and Breast Cancer Cell Lines: Molecular Mechanisms and Future Perspectives

**DOI:** 10.3390/molecules21010013

**Published:** 2015-12-22

**Authors:** Alina Uifălean, Stefanie Schneider, Corina Ionescu, Michael Lalk, Cristina Adela Iuga

**Affiliations:** 1 Department of Pharmaceutical Analysis, Faculty of Pharmacy, Iuliu Hațieganu University of Medicine and Pharmacy, Louis Pasteur Street 6, Cluj-Napoca 400349, Romania; alina.uifalean@umfcluj.ro; 2 Institute of Biochemistry, Ernst-Moritz-Arndt-University, Felix-Hausdorff Street 4, Greifswald 17487, Germany; stefanie.schneider1@uni-greifswald.de (S.S.); lalk@uni-greifswald.de (M.L.); 3 Department of Pharmaceutical Biochemistry and Clinical Laboratory, Faculty of Pharmacy, Iuliu Hațieganu University of Medicine and Pharmacy, Louis Pasteur Street 6, Cluj-Napoca 400349, Romania; corina.ionescu@umfcluj.ro

**Keywords:** breast cancer, genistein, isoflavone, estrogen receptor, molecular mechanism

## Abstract

The potential benefit of soy isoflavones in breast cancer chemoprevention, as suggested by epidemiological studies, has aroused the interest of numerous scientists for over twenty years. Although intensive work has been done in this field, the preclinical results continue to be controversial and the molecular mechanisms are far from being fully understood. The antiproliferative effect of soy isoflavones has been commonly linked to the estrogen receptor interaction, but there is growing evidence that other pathways are influenced as well. Among these, the regulation of apoptosis, cell proliferation and survival, inhibition of angiogenesis and metastasis or antioxidant properties have been recently explored using various isoflavone doses and various breast cancer cells. In this review, we offer a comprehensive perspective on the molecular mechanisms of isoflavones observed in *in vitro* studies, emphasizing each time the dose-effect relationship and estrogen receptor status of the cells. Furthermore, we present future research directions in this field which could provide a better understanding of the inner molecular mechanisms of soy isoflavones in breast cancer.

## 1. Introduction

In the 2014 statistics, breast cancer, together with lung and bronchial, and colorectal cancer, were estimated to be the three most commonly diagnosed types of cancer, accounting for one-half of all cancer cases in women. Breast cancer alone was expected to account for 29% (232,670) of all new cancers among women [1].

According to the American Society of Clinical Oncology, approximately 60% to 75% of women with breast cancer have estrogen receptor–positive breast cancer and 65% of these cancers are also progesterone receptor (PR) positive [2]. Multiple lines of evidence support the fact that the estrogen receptor (ER) signaling pathway is the major driver in stimulating proliferation, survival and invasion of breast cancer cells [3]. The assessment of ER expression is recommended in both early breast cancers and metastatic stages. Any detectable ER and/or PR expression (≥1%) using immunohistochemistry classifies these tumors as hormone receptor-positive [4]. The importance of ER status lies within its prognostic value, as it identifies patients most likely to benefit from endocrine forms of therapy.

Although blocking the activity of estrogen receptors has led to a considerable decline in breast cancer mortality [5], many patients become resistant to this therapy and develop metastatic tumors. Once metastases occur, the malignancy remains largely incurable, with a 5-year relative survival of 23% for distant stage diseases [6]. 

In general, the prevalence of breast cancers is lower in Asian than in North American and European countries, as epidemiological studies have demonstrated [7]. However, after migration to North America and Europe, breast cancer incidence in Asians increases and eventually equals the rates of the host country [8]. These statistics suggest that environmental factors, particularly dietary patterns, may play an important role in breast cancer development. As additional evidence of the role of one’s diet in cancer development, the breast cancer incidence and mortality has increased in Asian countries after a Western diet was adopted [8]. 

In the traditional Asian diet, soy foods are largely consumed, the daily intake of soy protein being estimated at 20–30 g (100 mg isoflavones). Conversely, a non-Asian diet contains less than 1 g of soy protein per day [9]. Due to these different food preferences, an inverse association between soy isoflavone intake and breast cancer risk has been demonstrated mostly for Asian populations and not for Western populations [10,11]. 

These observations have sparked a sustained interest in soy isoflavones as a promising therapeutic option in breast cancer chemoprevention. First of all, patients with increased breast cancer risk are taking into consideration supplementing their diet with soy or soy derivates, assuming that a high consumption might reduce the cancer risk [12]. After breast cancer diagnosis, American patients have reported dietary changes, adopting a higher soy intake, similar to the intake of vegetarians, but still less than that of Asian women [13]. In response to this growing demand, from 1996 to 2011, soy foods sales have increased from $1 billion to $5.2 billion [14]. Along with economic interests, soy isoflavones have generated great interest among scientists, for deciphering the cellular and molecular mechanisms underlying their potential chemopreventive role. 

Commonly, the chemopreventive role of soy isoflavones in breast cancer has been related to the interaction with estrogen receptors. However, recent studies have shown that the protective mechanisms of soy isoflavones are more intricate and yet not completely understood. In the present paper, we summarize the inhibitory effects of soy isoflavones on breast cancer cells and we provide a comprehensive view of the molecular mechanisms that underline their chemopreventive effects. 

## 2. Molecular Mechanisms of Soy Isoflavones

**Figure 1:**
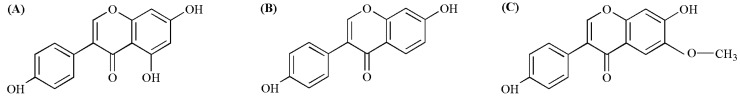
Chemical structures of soy aglycones: (**A**) genistein; (**B**) daidzein; (**C**) glycitein.

The predominant soy isoflavones are genistein, daidzein and glycitein which exist as glycosides, etherified glycosides and, to a lesser extent, as free forms also known as aglycones (Figure 1). These compounds present structural and functional similarities to 17-β-estradiol and can bind estrogen receptors alpha (ERα) and beta (ERβ). This explains their relationship to the phytoestrogen family, a class of non-steroidal phytochemicals which act like estrogen-like compounds [15]. 

Due to the structural resemblance with 17-β-estradiol, isoflavones mediate most of their biological effects through the modulation of estrogen-receptor signaling pathways. In hormone dependent tissues, estrogens play an important role in many physiological processes, such as cell proliferation, differentiation or apoptosis. However, high levels of estrogens are a major risk factor for the development of hormone-dependent diseases, such as breast or prostate cancer. It is still not completely clear why endogenous or synthetic estrogens increase breast cancer risk, while phytoestrogens, structurally similar compounds, appear to have the opposite effect.

Apart from ER-mediated signaling mechanisms, there is growing experimental evidence that soy isoflavones exert important ER-independent effects. Genistein has been shown to inhibit the growth of ER-negative breast cancer cells, demonstrating that other cellular mechanisms may play an important role in chemoprevention as well [16,17]. In fact, a pangenomic microarray analysis revealed that after genistein or daidzein exposure, there was only a partial overlap between the modulated molecular pathways in ER positive and ER negative cell lines [18]. Numerous *in vitro* studies have shown that isoflavones inhibit cell proliferation and trigger apoptosis by inhibiting the activity of several enzymes, such as tyrosine protein kinase [19,20], mitogen-activated kinase [17] or DNA topoisomerase II [20]. In addition to these, isoflavones, especially genistein, promote antioxidant defense and DNA repair [21,22], inhibit the development of tumor angiogenesis and metastasis [23] and also interfere in other ER-independent signal transduction pathways. 

It is difficult to make a clear distinction between estrogen dependent and independent mechanisms, as intrinsic cellular pathways often interfere or overlap. Isoflavone molecular mechanisms which are not ER-mediated can be investigated by several methods, either by knocking down the ER, blocking the ER using pure ER blockers or preferably, using ER negative breast cancer cell lines. 

## 3. ERs and GPER1 Mediated Mechanisms

The classical interaction of isoflavones with ER involves the binding to the ligand-binding domain of the receptor. Subsequently, the receptor-ligand complex binds to sequence-specific response elements known as estrogen response elements from DNA and the target gene transcription is then triggered. In addition to the classical genomic pathway, ERα and ERβ can also regulate gene transcription by rapidly activating Src/mitogen-activated protein (Src/MAP) kinase [24], phosphatidylinositide 3-kinases/Akt (PI3K/Akt) [25] and other direct DNA-binding transcription factors, such as activating protein 1 (AP1), specificity protein 1 (SP1), cAMP response element-binding protein (CREB), nuclear factor-κB (NF-κB) or p53 [26]. 

Although both ERα and ERβ are part of the steroid receptor superfamily, they are encoded by distinct genes (ESR1 and ESR2, respectively) and exert distinct biological functions. ERα is associated with aberrant proliferation, inflammation and the development of malignancy. By contrast, ERβ seems to oppose ERα actions on cell proliferation by modulating the expression of many ERα-regulated genes and exhibiting anti-migratory and anti-invasive properties in cancer cells [26]. 

The *in vitro* binding selectivity of soy isoflavones towards ERβ over ERα may provide insight into the biological activity of these natural compounds. Genistein presents 20 to 30-fold higher binding affinity for ERβ than for ERα, while daidzein has a 5-fold increased affinity for ERβ [27]. These binding capacities have been shown to vary considerably depending on the estrogenic endpoint used, especially for daidzein [28]. However, compared to the natural ligand, 17-β-estradiol, the binding affinity of isoflavones for ERα and ERβ is one to three orders of a lower magnitude [27,29]. Additionally, the active metabolite of daidzein, S-equol, shows a binding preference greater than that of its precursor and comparable to that of genistein. By contrast, the R isomer of equol exhibits a binding selectivity for ERα [30]. 

During tumor development, the ERα/ERβ balance is tilted in favor of ERα due to an upregulation of ERα mRNA levels within the tumor compartment [31]. As a consequence, the cellular response after isoflavones exposure is dependent not only on the receptor positivity, but also on the concrete ERα/ERβ expression level.

Using T47D breast cancer cell line with tetracycline-dependent ERβ expression and constant ERα expression, it has been shown that genistein can stimulate cell proliferation in the absence of ERβ expression. Additionally, in cells with full ERβ expression, genistein inhibited growth-stimulatory effects more efficiently than in cells with no expression of recombinant ERβ [29]. Also, depending on the ERα/ERβ ratio, isoflavones can influence cancer cell proliferation, apoptosis and cell cycle arrest as well [32]. Following genistein treatment, MCF-7 breast cancer cells (with high ERα/ERβ ratio) and MDA-MB-231 (ER negative) have shown an increased proliferation rate, while in T47D breast cancer cells (with low ERα/ER ratio), the same treatment produced cell cycle arrest, improved mitochondrial functionality [32] and decreased the oxidative stress [33]. 

To get deeper insight into the role of ERα/ERβ ratio, a global gene expression profile was performed on MCF-7 and T47D breast cancer cells exposed to soymilk extracts. At high ERα levels, soy isoflavones determined the same expression changes as those induced by estrogen, promoting the upregulation of multiple factors involved in the cell cycle, DNA replication, chromosome segregation and inhibition of apoptosis. When an inducible promoter was used to reconstitute the expression of ERβ, an attenuation of cell division growth-promoting factors was observed, along with a stimulation of cell proliferation arrest factors [34]. 

Furthermore, transcriptomics and stable isotope labeling by amino acids in cell culture (SILAC)-based proteomics were performed on T47D-ERβ breast cancer cells exposed to genistein. Results revealed that, in cells expressing ERβ, genistein decreased cell proliferation, induced cell cycle arrest and apoptosis. In the presence of ERβ, genistein reduced the cell motility and the metastatic potential, while ERα expression was correlated with cell proliferation [35]. Similar results were obtained after hierarchical clustering analysis based on transcriptomics data. The overlap of estrogen regulated genes was greater for genistein and equol, compared to the gene expression patterns of other phytoestrogens. Particularly, isoflavones had less stimulatory effects on proliferation, motility and inflammation genes compared to estrogen [36]. 

Thus, soy isoflavones, especially genistein, mediate important cellular processes via estrogen receptors and the ERα/ERβ ratio of the cell lines should be carefully considered when drawing conclusions. Although the binding affinity of genistein is higher for ERβ, there are particular conditions where genistein could lead to detrimental effects. Such are the cases of low to higher grades of ductal cancers and high-grade lobular cancers, characterized by loss of ERβ expression, high ERα level and high proliferation [37]. To these patients, soy consumption should be re-evaluated and special attention should be paid to the phytoestrogen daily intake.

Despite the structural similarity to 17-β-estradiol, isoflavones elicit not only estrogenic, but also antiestrogenic effects. Preclinical evidence has shown that at premenopausal levels of 17-β-estradiol (1 nM), isoflavones exert their effects as estrogen antagonist, while under low estrogen conditions, comparable to postmenopausal levels (0.01 nM), isoflavones act as estrogen agonist [38]. On the contrary, recent *in vitro* research has found that phytoestrogens induce proliferation of ER positive breast cancer cells at physiological concentrations of estrogen, but inhibit the growth and induce apoptosis in cells unexposed to estrogen [39] or in long-term estrogen-deprived cells [40]. In this light, the growth medium composition is particularly important for cells that express ERs, such as MCF-7. The ubiquitous pH indicator, phenol-red, has been shown to exert significant estrogenic activity at concentrations of 15–45 μM, such levels being found in culture media [41]. Thus, for reproducing a completely estrogen deprived medium, cells must be cultivated in a phenol red free medium which can be supplemented with charcoal stripped serum or with a synthetic serum. 

In explaining the heterogeneity of results, another essential factor is the considered isoflavone dose. Genistein mediates estrogenic effects and promotes cell growth at low concentrations (0.01–10 μM), a concentration of 100 nM producing proliferative effects similar to those induced by 1 nM estradiol [42]. On the contrary, higher concentrations of genistein (>20 μM) generate anti-estrogenic effects and inhibit cell growth [42,43,44,45,46]. In ER negative cells, this dual effect was not observed, genistein producing only antiproliferative effects, especially at high doses [47,48]. This suggests that the proliferative effects of genistein, observed at low doses, are ER mediated, while the antiproliferative effects, mainly observed at high doses are ER independent. However, this does not exclude the possibility that genistein can exert additional antiproliferative effects, ER mediated, especially in cells with high ERβ expression, as explained above. 

**Figure 2:**
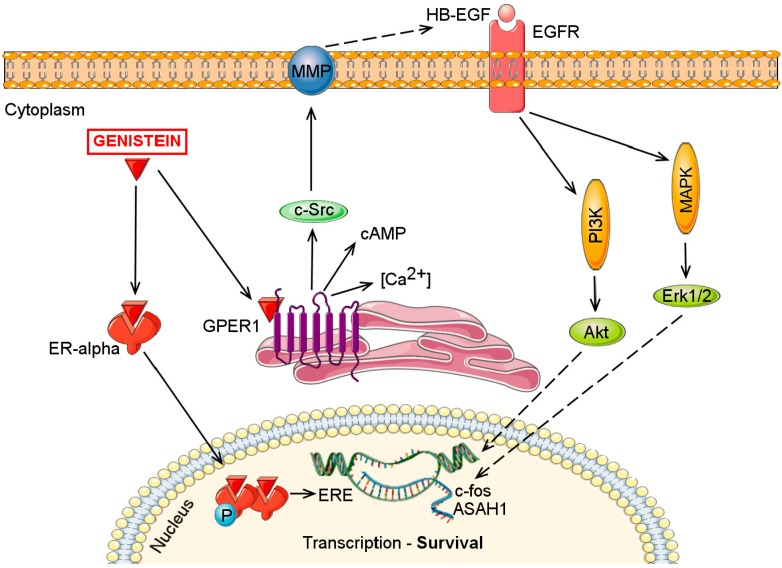
Genistein (0.01–10 μM) stimulates breast cancer cell proliferation acting through the classical genomic ER pathway and/or through the fast, non-genomic GPER1-mediated pathway. Abbreviations: Akt, Protein kinase-B; ASAH1, N-acylsphingosine amidohydrolase; cAMP, cyclic adenosine monophosphate; EGFR, Epidermal growth factor receptor; ER-alpha, Estrogen receptor alpha; ERE, Estrogen response elements; Erk1/2, Extracellular-signal-regulated kinase 1/2; GPER1, G protein coupled estrogen receptor 1; HB-EGF, Heparin-binding EGF-like growth factor; MAPK, mitogen-activated protein kinase; MMP, Matrix metalloproteinase; PI3K, Phosphoinositide 3-kinase.

The stimulatory effects of genistein have not been exclusively related to ERα interaction. Similar to 17-β-estradiol, genistein can induce cell proliferation via G protein coupled estrogen receptor 1 (GPER1) [49,50], as an alternative, non-genomic signaling pathway. Activation of GPER1 stimulates cAMP production, intracellular Ca^2+^ mobilization and induces c-Src activation. Subsequent, the transactivation of the epidermal growth factor receptor is triggered and downstream signaling pathways such as PI3K/Akt and mitogen-activated protein kinase/extracellular signal-regulated kinase (MAPK/ERK) are activated. Through GPER1-dependent pathways, genistein stimulated c-fos expression even in the absence of ERs [49] and stimulated acid ceramidase gene (ASAH1) expression in MCF-7 cells [50]. Thus, low concentrations of genistein can induce proliferation in breast cancer cells by modulating both the genomic and the non-genomic signaling pathways (Figure 2).

## 4. Effects on Apoptosis

In maintaining a healthy balance between cell survival and cell death, apoptotic mechanisms play important roles. Dysregulation of the apoptotic processes can allow neoplastic cells to survive over intended lifespans and favor treatment resistance to conventional therapies, requiring higher doses of cytotoxic agents. 

The apoptotic mechanisms of isoflavones have been widely investigated, *in vitro* studies reporting pro-apoptotic effects mainly through mitochondrial dependent pathways. Along with the ER status, the caspase-3 status represents another critical determinant in explaining the different responses of breast cancer cells towards isoflavone treatment. Transfection of caspase-3 in MCF-7 cells resulted in enhanced apoptotic death after genistein exposure, while caspase-3 knockdown in MDA-MB-231 cells rendered cells to be more resistant to genistein [51]. Among other phytoestrogens, genistein and equol also activated caspase-4, which binds interleukin 6 (IL-6), a proinflammatory cytokine, inducing an inflammatory stress response to the cells [40]. 

In MCF-7 cells, equol and 4-hydroxy-tamoxifen (4-OHT), the active metabolite of tamoxifen, induced activation of caspase-9 and caspase-7, together with cytochrome-c release into cytosol. The combination of these two induced a more potent inhibition than individual exposure. Following treatment with Z-VADFMK, a pan-caspase inhibitor, inhibited equol- and 4-OHT-mediated apoptosis, indicating that apoptosis is mainly caspase-mediated. These effects were observed after relatively high doses of equol (100 μM) and 4-OHT (10 μM) [52]. In a similar experiment, daidzein (25–100 μM) induced cell death in a dose dependent manner acting through the same mitochondrial pathways [53].

Some reports suggest that genistein-induced caspase-7 activation involves the activation of calpain by Ca^2+^ depletion of the endoplasmic reticulum [54]. Moreover, genistein induces DNA damage-inducible transcript 3 (DDIT3), a marker of endoplasmic reticulum stress associated with cell death and of inositol requiring protein 1 alpha (IRE1α), an unfolded-protein-response sensor, which is activated to relieve stress [40]. 

Numerous investigators have also reported that genistein induces apoptosis through downregulation of Bcl-2, Bcl-xL and upregulation of Bax or by releasing cytochrome C into the cytosol [17,22,48,53,55]. In ER negative MDA-MB-231 cells, low doses of genistein (1 μM) increased the Bax/Bcl-2 ratio along with a significant decrease in phosphorylated Extracellular signal Regulated Kinase 1/2 (ERK1/2), but only in the presence of 1 nM 17-β-estradiol. Higher genistein concentrations (100 μM) stimulated apoptosis independent of 17-β-estradiol presence through mechanisms that were not correlated with the Bax/Bcl-2 ratio or with phosphorylation of ERK1/2 [50]. So, it is possible that low doses of genistein induce cell death through Bax/Bcl-2 pathways, but higher concentrations lead to cell death through other cytotoxic mechanisms. 

Altogether, genistein exerts apoptotic effects mainly by caspase activation, the activation of several endoplasmic reticulum stress regulators and Bax/Bcl-2 ratio upturn. Additional mechanisms have also been advanced, such as the inhibition of the proteasome activity [56] or the downregulation of anti-apoptotic survivin [22]. In most cases, apoptotis is induced after high concentrations of genistein or daidzein, above 50 μM [22,52,57]. As genistein triggers apoptosis also in ER negative cell lines [17,48] or after ERα knockdown [55], some apoptotic mechanisms might not require the ER expression. 

## 5. Effects on Cell Proliferation and Survival

### 5.1. Inhibition of NF-κB Pathway Activation

Activation of NF-κB is confined predominantly to inflammatory and ER-negative breast cancer subtypes, but constitutive NF-κB activity has also been observed in ER positive cancer types [58,59]. In fact, the progression to a more aggressive, endocrine-resistant breast cancer phenotype can be attributed to a positive cross-talk between ER and NF-κB activation, suggesting that these two transcription factors cooperate to upregulate the expression of several genes involved in cell survival and chemoresistance [60]. 

Soy isoflavones have shown to inhibit NF-κB activation, blocking mainly the canonical NF-κB activation pathway. In MCF-7 breast cancer cells engineered to overexpress oncogenic HER2 (MCF-7 HER2) and control vector cells (MCF-7 vec), genistein (100 μM) inhibited the phosphorylation of IκBα, sequestering NF-κB complexes into cytoplasm [61]. Similar results were obtained for MDA-MB-231 cells, where lower doses of genistein (5–20 μM) caused a concentration-dependent decrease in NF-κB/p65 nuclear protein levels, most likely by inhibiting the phosphorylation of IκB proteins [17]. Moreover, genistein inhibited the translocation of NF-κB dimers to the nucleus and their binding to DNA, preventing the transcription of NF-κB downstream genes [17,61]. 

The inhibition of NF-κB activity by genistein can be mediated via Notch-1, a signaling pathway with an important regulatory role in triple negative breast cancers. In MDA-MB-231 cells, genistein (≥20 μM) inhibited Notch-1 expression together with the downregulation of NF-κB targeted proteins: cyclin B1, Bcl-2 and Bcl-xL. As the downregulation of Notch-1 and NF-κB expression by siRNA inhibited the expression of these proteins, it was suggested that NF-κB inactivation is mediated via Notch-1 pathway [16]. 

Both NF-κB and Notch-1 pathways are mainly expressed in triple negative breast cancer, the cancer subtype with the worst prognosis among all breast cancer subtypes [62]. As there is no targeted therapy for this subtype, genistein could represent a therapeutic option in blocking both NF-κB and Notch-1 pathways.

### 5.2. Effects on PI3K/Akt/mTOR Signaling Pathway

The phosphatidylinositol 3-kinase/protein kinase-B mammalian target of rapamycin (PI3K/Akt/mTOR) intracellular pathway plays a crucial role in cellular survival, proliferation or protein synthesis. Hyperactivation of this pathway has been associated with tumor development and resistance to anticancer therapies [63]. In breast cancers, PI3K/Akt/mTOR is the most frequently activated signaling pathway [64], with more than 70% of breast cancers presenting molecular alterations in one or more components of the PI3K/Akt pathway [65]. A significant cross-talk between the PI3K/Akt/mTOR and the ER pathway has been established, PI3K/Akt/mTOR inhibition expanding the endocrine therapy benefit in ER positive breast cancers, from the first-line setting and beyond [64].

There is strong evidence that the activation of PI3K/Akt pathway can take place through insulin-like growth factor 1 receptor (IGF-1R). At high doses (≥20 μM), genistein has shown to inhibit the activation of the IGF-1R/Akt signaling pathway, leading to apoptosis through downregulation of Bcl-2 and upregulation of Bax [66]. Contrarily, at low doses (1 μM), genistein mimics the estrogen stimulatory effects, increasing the mRNA expression of the IGF-1R. When cells were co-treated with JB-1, an IGF-1R antagonist, this effect was completely blocked [67]. These results uphold again the dose dependent effect of genistein and the fact that inner mechanisms could rely on IGF-1R interaction and the subsequent activation of PI3K/Akt pathway. 

In the PI3K/Akt cascade, genistein can also act on a more downstream level, inducing the expression of PTEN, the natural inhibitor of PI3K/Akt signaling pathway. Using a non-tumorigenic human mammary epithelial cell line, MCF-10A, genistein increased PTEN and p53 expression. Next, a sequence of PTEN-dependent reactions is triggered, initiating an autoregulatory loop between PTEN and p53 that can stimulate mammary epithelial cell cycle arrest and early lobuloalveolar differentiation. Notably, genistein’s stimulatory effects on PTEN and p53 occurred at low genistein doses (2 μM), that correspond to serum concentrations of regular soy consumers [68]. 

The decreased PTEN level could be attributed to homeobox transcript antisense RNA (HOTAIR) oncogenic effects, as was demonstrated for laryngeal squamous carcinoma cells. HOTAIR promoted PTEN methylation resulting in a loss of PTEN expression and seizing the opportunity for PI3K/Akt activation [69]. In breast cancer, HOTAIR plays also a promoter role, overexpression of HOTAIR being associated with metastasis and poor overall survival [70]. Recently, it has been shown that calycosin or genistein (80 μM) reduced HOTAIR expression and decreased phosphorylation of Akt in MCF-7 cells [71]. 

All the above studies have evaluated genistein’s influence on the growth of breast cancer cell lines after a short-term exposure, 48 or 72 h. Considering that short time exposure to genistein does not reflect the long term effects induced by a soy diet, a different *in vitro* experimental model tried to determine the effects of low-dose, long-term genistein exposure. For this, ER expression and PI3-K/Akt signaling activity were assessed after MCF-7 cells were treated with 10 nM genistein for 10–12 weeks. Long-term genistein treatment reduced the growth promoting effects of estrogen, although there was no change in the ERα expression. Also, genistein decreased the protein expression of total Akt and phosphorylated Akt and increased the ability of a PI3-kinase inhibitor, LY 294002, to suppress cell growth. As a result of the above mentioned, long-term genistein treatment could alter the PI3-K/Akt signaling pathway in ER positive breast cancer cells [72]. 

Overall, genistein can interfere at several levels in the PI3-K/Akt cascade, either by blocking the IGF-1R or stimulating the inhibitory effects of PTEN, or, at a lower level, by reducing the protein expression of total Akt and phosphorylated Akt. To our knowledge, a direct correlation between genistein exposure and inhibition on PI3-K/Akt via its downstream target, mTOR, has not been established for breast cancer cells until now. Still, downregulation of mTOR expression after isoflavone treatment has been demonstrated for other types of hormonal cancers, such as prostate or ovary [73].

### 5.3. Effects on MAPK/ERK Signaling Pathway

Targeting only the PI3K/Akt/mTOR signaling pathway does not guarantee that the survival signals will not be transmitted to the downstream nuclear effectors. In fact, inhibition of PI3K/Akt/mTOR proved the activation of another important pathway dysregulated in breast cancer, the mitogen-activated protein kinase/extracellular signal-regulated kinase (MAPK/ERK) pathway [74]. Activation of these pathways through hetero-dimerization of erbB2 and erbB3 can lead to multi-drug resistance in breast cancers [75]. As there are multiple points of convergence, cross-talk and feed-back loops between these two signaling pathways, finding a joint inhibitor could lead to a greater inhibitory effect [74]. 

Genistein has been shown to inhibit the MAPK signaling pathway in both ER positive and ER negative breast cancer cells, but apparently through different mechanisms. In MDA-MB-231 cells, genistein (5–20 μM) suppressed the protein levels of MEK5, total ERK5 and phospho-ERK5 in a dose-dependent manner [17]. In MCF-7 cells, high genistein concentrations (100 μM) triggered apoptosis by activating the p38 MAPK through Ca^2+^ release from the endoplasmic reticulum [54].

**Figure 3:**
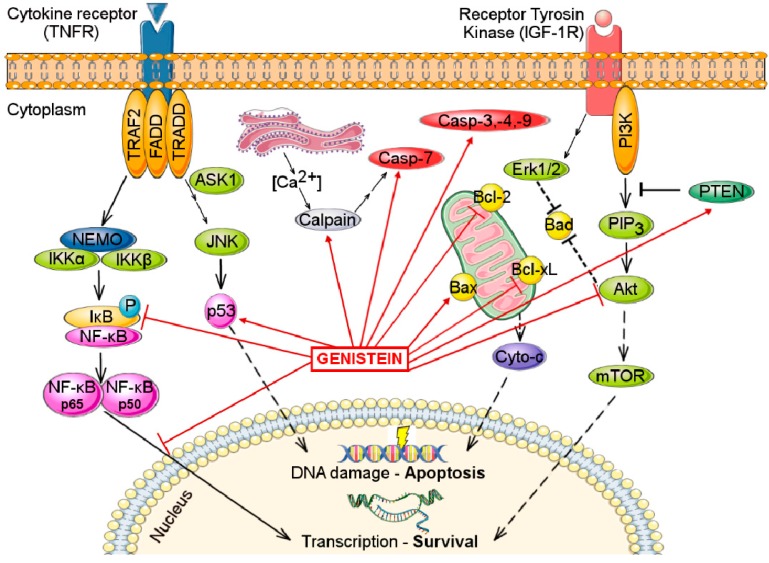
The main molecular targets of genistein that are involved in apoptosis and cell survival mechanisms. Arrow-headed lines indicate activation (or upregulation) and bar-headed lines indicate inhibition (or downregulation). Abbreviations: Akt, Protein kinase-B; ASK1, Apoptosis signal-regulating kinase 1; Bad, Bcl-2-associated death promoter; Bcl-2, B-cell lymphoma 2; Bcl-xL, B-cell lymphoma-extra large; Casp-3,-4,-9, Caspases 3, 4 and 9; Cyto-c, Cytochrome-c; Erk1/2, Extracellular-signal-regulated kinase 1/2; FADD, Fas-Associated protein with Death Domain; IKKα and IKKβ, IκB kinases; JNK, Jun amino-terminal kinases; mTOR, mammalian target of rapamycin; NEMO, NF-κB essential modulator; NF-κB, Nuclear factor κB; p53, Tumor protein p53; PI3K, Phosphoinositide 3-kinase; PIP_3_, Phosphatidylinositol (3,4,5)-trisphosphate; PTEN, Phosphatase and tensin homolog; TRADD, TNF receptor-associated death domain; TRAF2, TNF receptor-associated factor 2.

As discussed herein, genistein can also induce cell growth, at concentrations below 10 μM. The stimulatory effects of genistein can also be attributed to delayed and prolonged phosphorylation of ERK1/2. Co-incubation of MCF-7 cells with an ERK inhibitor abolishes ERα transactivation, indicating that the MAPK/ERK signaling pathway is necessary for ER-mediated transcription [76]. The same stimulatory effects were observed in erbB-2-transfected ER positive MCF-7 cells treated with low doses of genistein. These effects were due to the enhanced activation of ER, MAPK/ERK1/2 and PI3K/Akt signaling pathways, underlying the close ER—erbB-2 cross-talk in breast cancer cells [77]. Therefore, isoflavones and especially genistein, can interfere in several pathways that control apoptosis and cell survival, targeting key molecules, as depicted in Figure 3.

## 6. Effects on Angiogenesis and Metastasis

Pathological angiogenesis is a sequential process characterized by a shift between pro-angiogenic and anti-angiogenic factors. It is usually triggered by a hypoxic microenvironment that will activate various oxygen sensors, growth factors, angiopoietins, junctional molecules, endothelial sensors, finally leading to enhanced vascularization and rapid tumor growth [78]. Following angiogenesis, cancer cells are allowed to spread and invade nearby tissues, creating metastases. For breast cancer, bone metastases represent the most common metastatic site overall or the exclusive first site of metastasis. Lung, liver and brain are the next most common sites, in descending order of incidence [79]. 

Soy isoflavones, and particularly genistein, have been intensively examined for their anti-angiogenetic properties using endothelial cell lines [23,80,81]. The proposed mechanisms are mostly related to inhibition of vascular endothelial growth factor/basic fibroblast growth factor (VEGF/bFGF), as the VEGF family is widely recognized as a key regulator in tumor angiogenesis. Generally, the anti-angiogenetic effects were observed at medium and high concentrations of soy isoflavones (10–150 μM). In return, lower doses (0.1–10 μM) of genistein increased VEGF secretion in MCF-7 (ER positive), MELN (derived from MCF-7 cells) and MELP (derived from MDA-MB-231 cells and transfected with ERα), but not in MDA-MB-231 cells (ER negative), suggesting that ERα is necessary for VEGF stimulation [82]. 

The same dual effect of genistein was observed for the C-X-C chemokine receptor type 4 (CXCR4) and C-X-C motif chemokine 12 (CXCL12) levels in breast and ovarian cancer cells. The interaction between CXCR4 and CXCL12 plays an important role in cancer progression, adhesion and metastasis. The exposure to >10 μM genistein downregulated CXCR4, inhibiting chemotaxis and chemoinvasion of breast and ovarian cancer cells towards CXCL12. Then again, low doses of genistein (1–10 μM) upregulated CXCL12 mRNA levels in MCF-7 cells, proving once again the twofold effect of genistein [83]. Similar conclusions were drawn after an oligonucleotide microarray experiment, where genistein (30 μM and 50 μM) downregulated the expression of CXCL12 and matrix metalloproteinase 2 (MMP-2) and 7 (MMP-7). At the same time, genistein upregulated several invasion and metastasis inhibitors, such as tissue factor pathway inhibitor-2 (TFPI-2), activating transcription factor 3 (ATF3), DNA methyltransferase 1 (DNMT1) and membrane-type 1 matrix metalloproteinase cytoplasmic tail-binding protein-1 (MTCBP1) genes [84]. Inhibition of invasion via MMP-2 downregulation was also observed in MDA-MB-231 cells after treatment with daidzein or equol enantiomers (50 μM) [85]. For the same cell line, the activity of MMP-3 remained unaffected after genistein, genistin or daidzein treatment (10–30 μM) [86]. 

A recent experimental model of murine mammary cancer 4T1 cells engineered with luciferase has shown that soy isoflavones (<10 µM) had limited effects on the growth, motility or invasion of 4T1 cells *in vitro*. However, after the cells were injected into the tibia of female Balb/c mice, they stimulated metastatic tumor formation and increased Ki-67 protein expression [87]. The stimulatory effect observed *in vivo* could be due to systemic effects between the host, 4T1 tumors and soy isoflavones.

In summary, genistein (>10 μM) can exhibit anti-angiogenic and anti-metastatic effects in breast cancer cells through multiple mechanisms. These mechanisms involve the downregulation of VEGF and other pro-angiogenic factors and also the downregulation of MMPs and upregulation of angiogenesis inhibitors. But the modulatory mechanisms of genistein in breast cancer are far from being fully identified. In prostate cancer cells, it has been proved that genistein inhibits expression/accumulation of other pro-angiogenic factors like the hypoxia-inducible factor-1α (HIF-1α), apurinic apyrimidinic endonuclease redox effector factor-1 (APE1/Ref-1) or interleukin-8 (IL-8) [88]. Further studies are required to find out whether genistein interferes with these molecules in breast cancer cells as well, or if the mechanisms are tissue-specific. 

## 7. Effects of Soy Isoflavones on Reactive Oxygen Species and DNA Damage

Excessive generation of reactive oxygen species (ROS) has been linked to breast cancer development, progression and resistance to therapy. ROS can induce epigenetic changes or activate several growth-promoting signaling pathways such as PI3K/Akt, ERK1/2, MAPK/ERK or EGFR, finally leading to mitochondrial dysfunction and DNA damage [89,90]. In ER–positive breast cancer cells as MCF-7, higher ROS levels and greater DNA damage are induced by estrogen through ER dependent mechanisms [91]. More precisely, the ERα/ERβ ratio determines the oxidative status in response to estrogen, as cells with high ERα/ERβ ratio showed increased oxidative damage along with low levels of antioxidant enzymes and uncoupling proteins [92]. 

Like estrogen, genistein modulates oxidative stress in breast cancer cell lines according to the ERα/ERβ ratio [32,33]. After treatment with genistein (1 μM), the low ERα/ERβ ratio T47D cells showed improved mitochondrial functionality and antioxidant enzyme activity, the upregulation of uncoupling protein 2 and sirtuins and overall, lower oxidative stress. In contrast, in MCF-7 cells, characterized by high ERα/ERβ ratio, genistein treatment did not cause any change in mitochondrial functionality, antioxidant response or sirtuins levels [33]. These distinct effects could be due to genistein’s greater affinity towards ERβ, as discussed above. In MCF-7 cells, a decreased expression of antioxidant enzymes, namely CuZnSOD, MnSOD and thioredoxin reductase (TrxR), along with the upregulation of glutathione peroxidase (GPx) expression was reported only after exposure to higher genistein doses (100 μM). This may favor oxidative stress formation with consequent apoptosis and autophagy induction [22].

Apart from the modulation of oxidant enzymes, genistein can also induce death to MDA-MB-231 cells through mobilization of endogenous copper ions and generation of reactive oxygen species. After genistein treatment (50 μM), the superoxide anion is generated, then rapidly converted to hydrogen peroxide (H_2_O_2_), which causes the formation of hydroxyl radical (HO^−^) through the oxidation of reduced copper, according to the Fenton reaction. As ROS accumulate, irreversible DNA damage occurs, leading to cell death [93]. Furthermore, genistein (5 μM) can act as a suppressor of cytochrome enzymes CYP1A1 and CYP1B1, reducing the oxidative DNA damage induced by polycyclic aromatic hydrocarbons in the normal breast cancer cell line, MCF-10A [21]. 

Altogether, genistein has been shown to modulate the oxidative status of breast cancer cells either by favoring ROS accumulation or by decreasing the antioxidant defense and, therefore, inducing cell death. In order to improve the antioxidant properties of genistein, structural modulation has been made towards increasing the number of hydroxyl groups. Thus, the bioconversion of genistein to 2′-hydroxygenistein has shown a superior radical scavenging activity and a greater antiproliferative effect on MCF-7 cells [94]. 

## 8. Conclusions and Perspectives

The mechanisms of soy isoflavones in breast cancer have conventionally been linked to the modulation of ER, especially ERβ. However, current *in vitro* studies show that soy isoflavones interfere in other signaling pathways that control cell progression, such as NF-κB, PI3K/Akt or MAPK/ERK. Moreover, isoflavones can initiate apoptotic events, inhibit angiogenesis signaling pathways or interfere in the redox state of the cells. 

Recently, several lines of evidence support the fact that isoflavones exert also epigenetic properties, reducing DNA methylation [95] or modulating the histone acetylation [96]. In addition, genistein has been shown to enhance the radiosensitivity of tumoral cells [97] and to increase the effect of chemotherapeutic agents such as doxorubicin [98] or trastuzumab [99]. These novel mechanisms must be further explored in close connection with the existing data in order to have an accurate overview of the results and explain the potential contradictory results. 

Furthermore, conclusions should be drawn only in relation to the cellular ER status and the ERα/ERβ ratio for ER positive cells. In order to mimic a particular physiological state (premenopausal or postmenopausal), prior estrogen exposure or depletion of the cells should be made. Special attention should be given to doses, as soy isoflavones exert dose-dependent effects. 

In addition to classical *in vitro* assays, novel high-throughput approaches, such as cell culture proteomics [35], transcriptomics [36] or metabolomics [100] are rapidly gaining ground. Advances in DNA microarrays, two-dimensional electrophoresis, labeling techniques, NMR and LC-MS techniques will provide a comprehensive overview of the inner molecular mechanisms of isoflavones in breast cancer cells. 

In parallel, considerable attention is given to improving isoflavones properties as well. As genistein has unsuitable physiochemical properties to drug formulation, a step forward has been made by designing genistein-loaded liposomes [101] and genistein-loaded biodegradable nanoparticles [102] with superior solubility, stability and drug delivery. Other studies have focused on enhancing the anticancer activity of genistein by modulating the structure-activity relationship. Synthetic structurally-modified derivatives of genistein were obtained by coordination with copper (II) [103], 2′-hydroxylation [94] or conjugation with polysaccharides [104,105] and exerted higher anti-cancer activity compared to parent genistein.

Novel approaches of cancer treatment are in favor of multi-target agents in order to reduce activation of compensatory mechanisms that lead to drug resistance. As soy isoflavones exert pleotropic effects and modulate multiple signaling pathways, they represent promising naturophatic agents for the management of breast cancers [106].

Everything considered, there is still a keen interest in exploring isoflavones chemopreventive properties as cellular mechanisms are not fully understood. Once the molecular mechanisms of isoflavones are addressed, *in vivo* experiments must be carried out in order to validate the preclinical results. Together, these studies will provide a deeper understanding of the role of isoflavones in breast cancer chemoprevention and chemotherapy.
